# Supplementary material for: Truncation and constitutive activation of the androgen receptor by diverse genomic rearrangements in prostate cancer
Source: Nat Commun. 2016 Nov 29;7:13668. doi: 10.1038/ncomms13668 (PMC5141345; doi:10.1038/ncomms13668)
Supplement: Supplementary Information — Supplementary Figures 1-14 and Supplementary Tables 1-9. [file ncomms13668-s1.pdf]

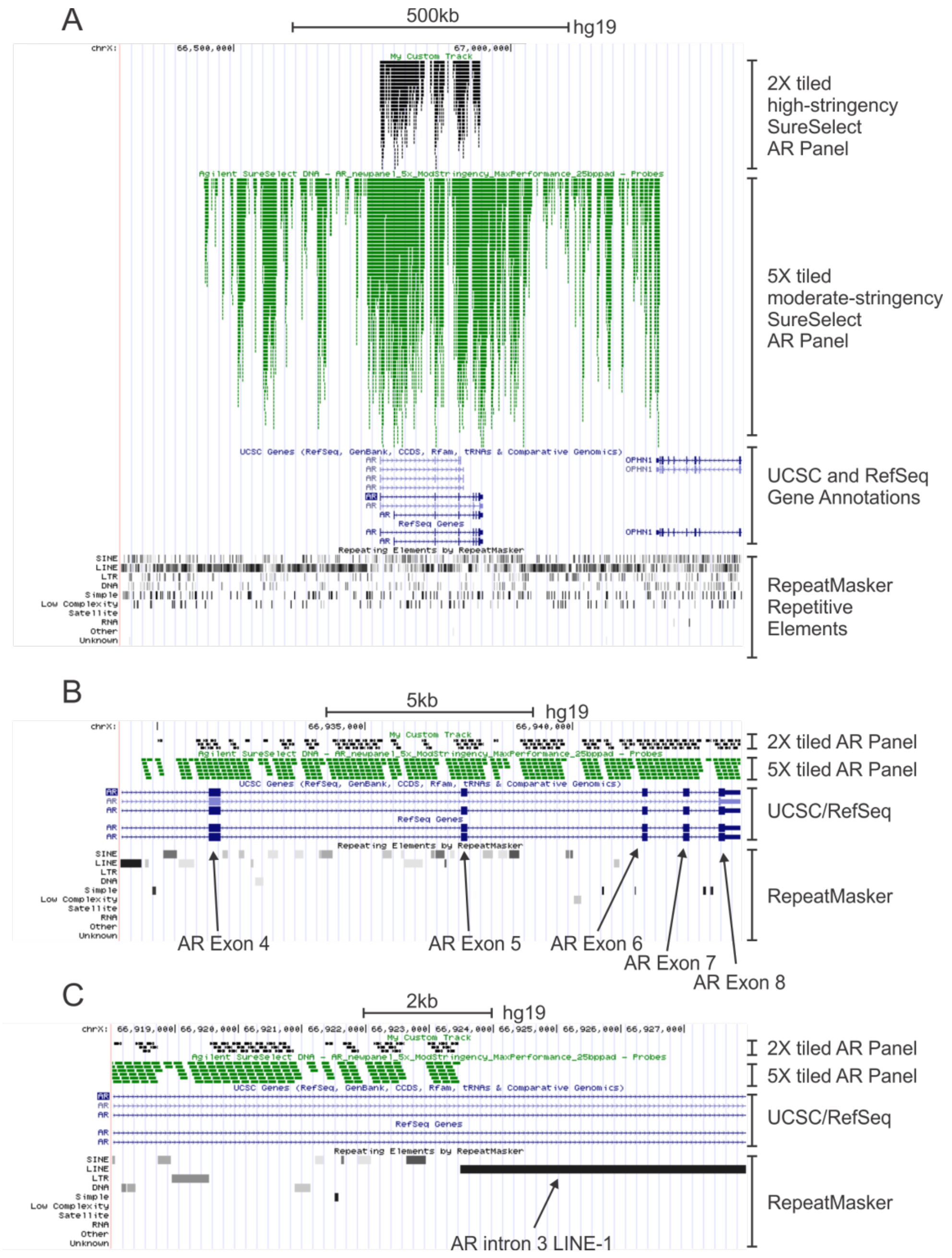

Supplementary Figure 1. Improvements in Agilent SureSelect AR capture array design. Visualization of AR capture libraries in BED format using the UCSC genome browser illustrates key improvements compared with a previous panel used to interrogate structural variants in the AR locus. These improvements are **A,** coverage extending upstream and downstream of the AR gene, **B,** 5X tiling of baits as opposed to 2X tiling, and moderate allowance for repetitive elements identified by RepeatMasker as having low sequence conservation (denoted by lighter gray boxes in the RepeatMasker track). **C,** despite these improvements, the AR capture array is still blind to highly-conserved repetitive elements including a LINE-1 in AR intron 3 (denoted by darker gray and black boxes in the RepeatMasker track).

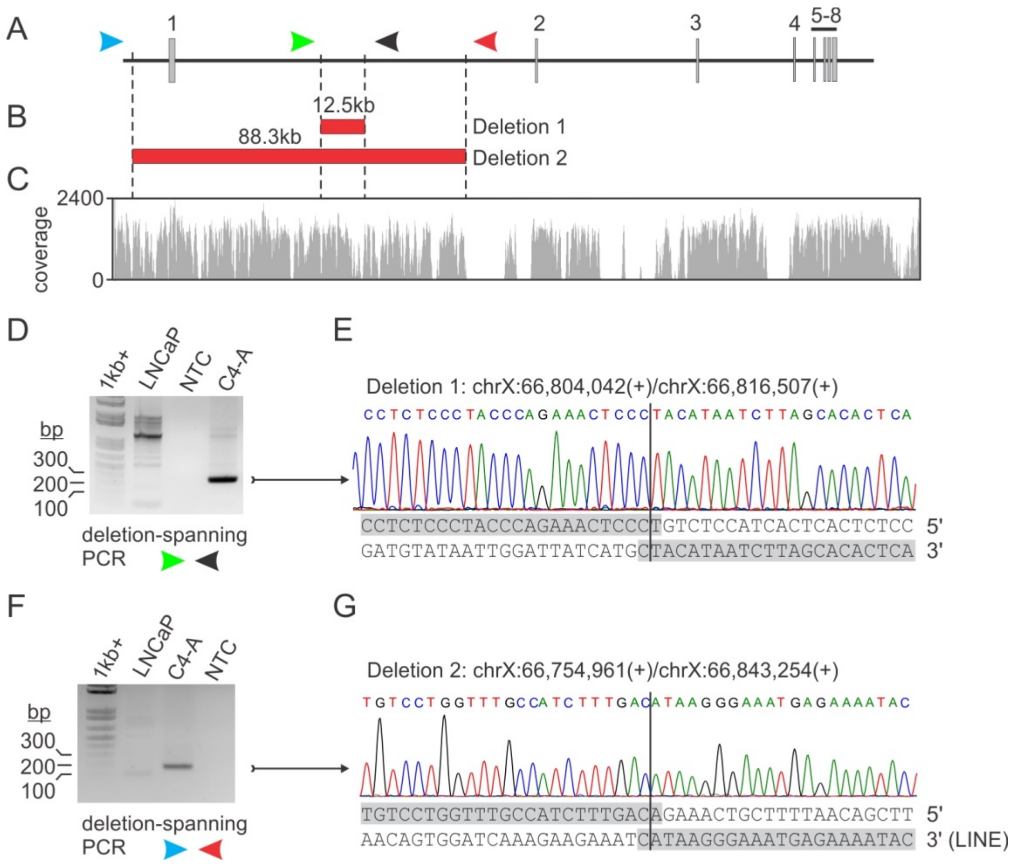

Supplementary Figure 2. *AR* genomic structural rearrangement validation in subject C-4. A and B, binding locations of PCR primers (colored arrowheads) for validation of Deletion 1 and Deletion 2 in tumor C-4A. **C,** AR DNA-seq coverage for tumor C-4A was assessed by visualizing BAM files of mapped AR DNA-seq reads in Integrative Genomics Viewer. **D,** PCR was performed with a primer set designed for isolation of Deletion 1 (illustrated in A and B) using DNA from tumor C-4A as well as LNCaP or no template control (NTC) as negative controls. **E,** Sanger sequencing analysis of the tumor C-4A Deletion 1 PCR product. The genomic sequences corresponding to the 5’ and 3’ breakpoints are indicated, with gray shading representing the sequences contained in the break fusion junction signature. **F,** PCR was performed with a primer set designed for isolation of Deletion 2 (illustrated in A and B) using DNA from tumor C-4A as well as LNCaP or no template control (NTC) as negative controls. **G,** Sanger sequencing analysis of the tumor C-4A Deletion 2 PCR product. The genomic sequences corresponding to the 5’ and 3’ breakpoints are indicated, with gray shading representing the sequences contained in the break fusion junction signature. The 3’ breakpoint from Deletion 2 is located within a LINE-1 element.

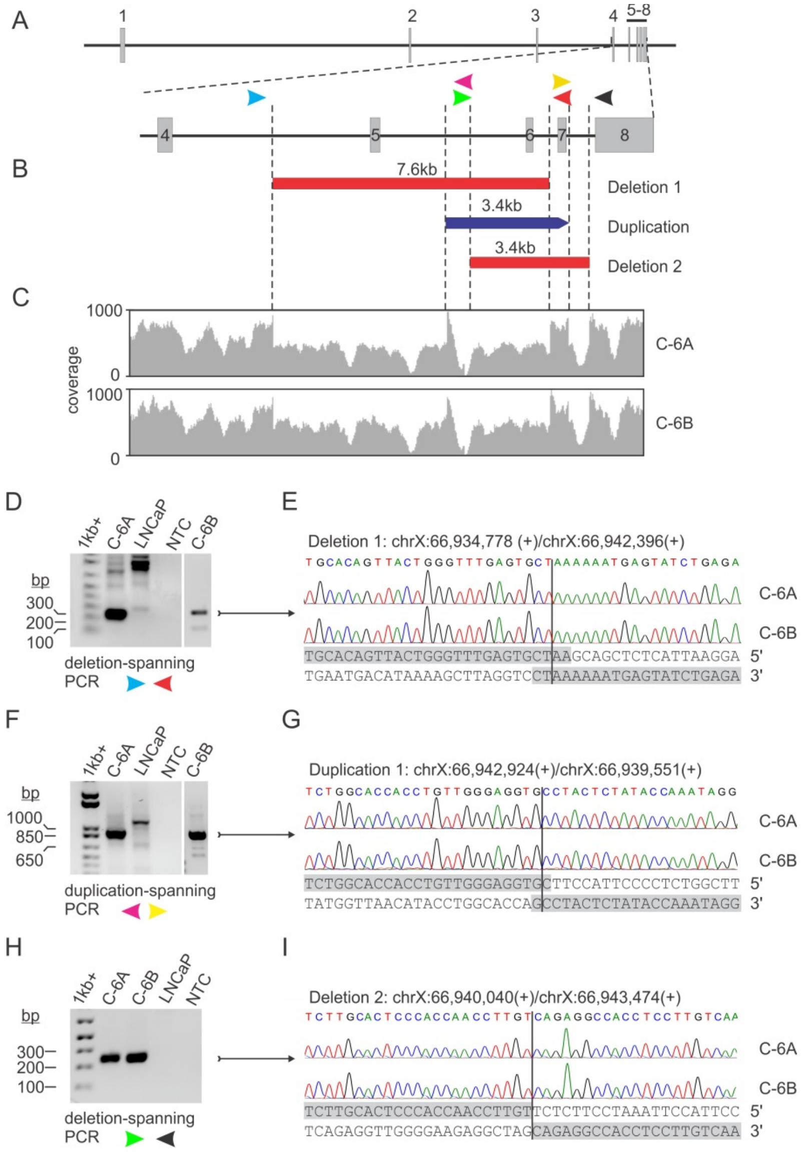

Supplementary Figure 3. ***AR*** **genomic structural rearrangement validation in subject C-6. A and B,** binding locations of PCR primers (colored arrowheads) for validation of Deletion 1, Duplication, and Deletion 2 in tumors C-6A and C-6B. **C,** AR DNA-seq coverage for tumors C-6A and C-6B was assessed by visualizing BAM files of mapped AR DNA-seq reads in Integrative Genomics Viewer. **D,** PCR was performed with a primer set designed for isolation of Deletion 1 (illustrated in A and B) using DNA from tumors C-6A and C-6B as well as LNCaP or no template control (NTC) as negative controls. **E,** Sanger sequencing analysis of the tumor C-6A and C-6B Deletion 1 PCR products. The genomic sequences corresponding to the 5' and 3' breakpoints are indicated, with gray shading representing the sequences contained in the break fusion junction signature. **F,** PCR was performed with a primer set designed for isolation of the Duplication (illustrated in A and B) using DNA from tumors C-6A and C-6B as well as LNCaP or no template control (NTC) as negative controls. **G,** Sanger sequencing analysis of the tumor C-6A and C-6B Duplication PCR products. The genomic sequences corresponding to the 5' and 3' breakpoints are indicated, with gray shading representing the sequences contained in the break fusion junction signature. **H,** PCR was performed with a primer set designed for isolation of Deletion 2 (illustrated in A and B) using DNA from tumors C-6A and C-6B as well as LNCaP or no template control (NTC) as negative controls. **I,** Sanger sequencing analysis of the tumor C-6A and C-6B Deletion 2 PCR products. The genomic sequences corresponding to the 5' and 3' breakpoints are indicated, with gray shading representing the sequences contained in the break fusion junction signature.

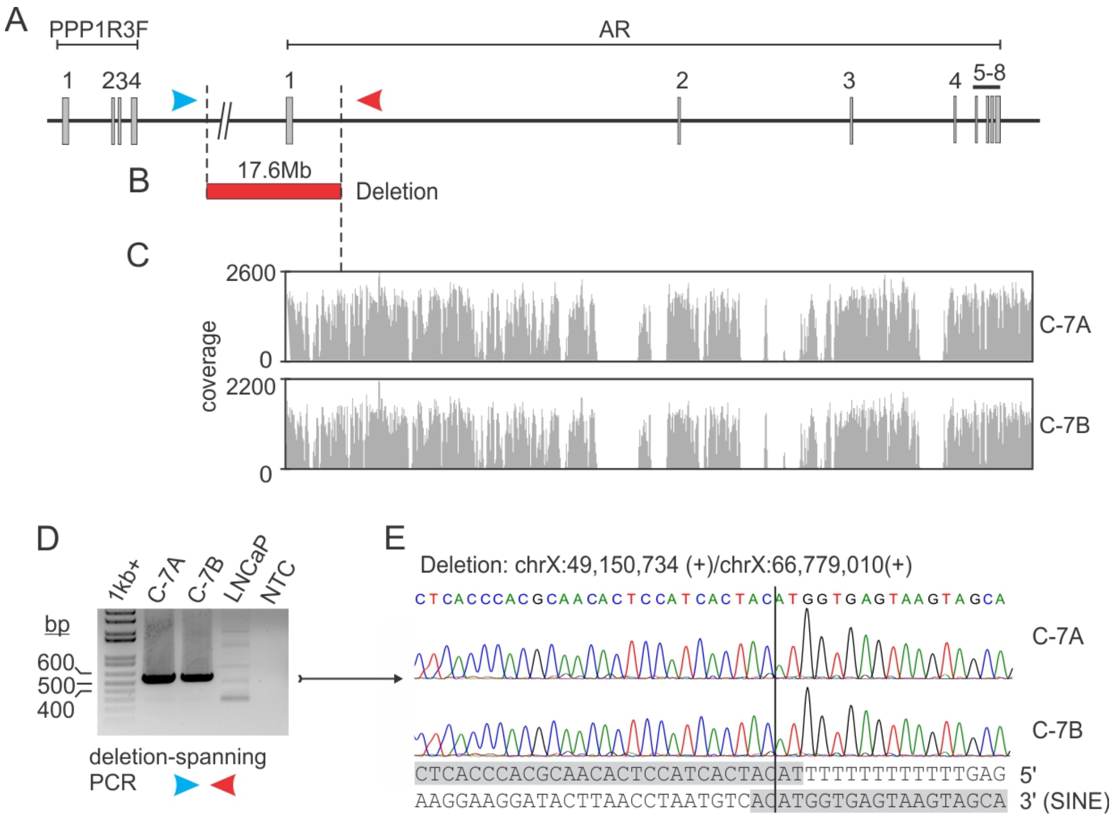

Supplementary Figure 4. *AR* genomic structural rearrangement validation in subject C-7. A and B, binding locations of PCR primers (colored arrowheads) for validation of the Deletion in tumors C-7A and C-7B. **C,** AR DNA-seq coverage for tumors C-7A and C-7B was assessed by visualizing BAM files of mapped AR DNA-seq reads in Integrative Genomics Viewer. **D,** PCR was performed with a primer set designed for isolation of the Deletion (illustrated in A and B) using DNA from tumors C-7A and C-7B as well as LNCaP or no template control (NTC) as negative controls. **E,** Sanger sequencing analysis of the Deletion PCR products from tumors C-7A and C-7B. The genomic sequences corresponding to the 5' and 3' breakpoints are indicated, with gray shading representing the sequences contained in the break fusion junction signature The 3' breakpoint sequence is located within a SINE element.

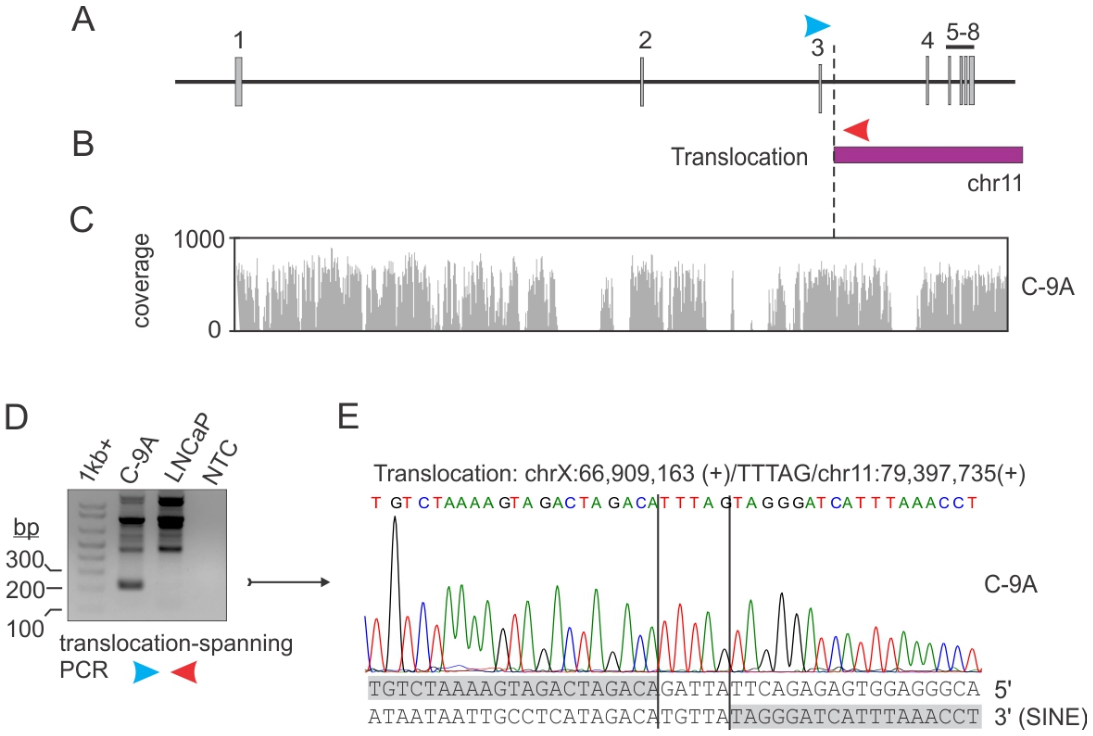

Supplementary Figure 5. ***AR*** **genomic structural rearrangement validation in subject C-9. A and B,** binding locations of PCR primers (colored arrowheads) for validation of the Translocation in tumor C-9A. **C,** AR DNA-seq coverage for tumor C9-A was assessed by visualizing BAM files of mapped AR DNA-seq reads in Integrative Genomics Viewer. **D,** PCR was performed with a primer set designed for isolation of the Translocation (illustrated in A and B) using DNA from tumor C9-A as well as LNCaP or no template control (NTC) as negative controls. **E,** Sanger sequencing analysis of the Translocation PCR product from tumor C9-A. The genomic sequences corresponding to the 5' and 3' breakpoints are indicated, with gray shading representing the sequences contained in the break fusion junction signature The 3' breakpoint sequence is located within a SINE element.

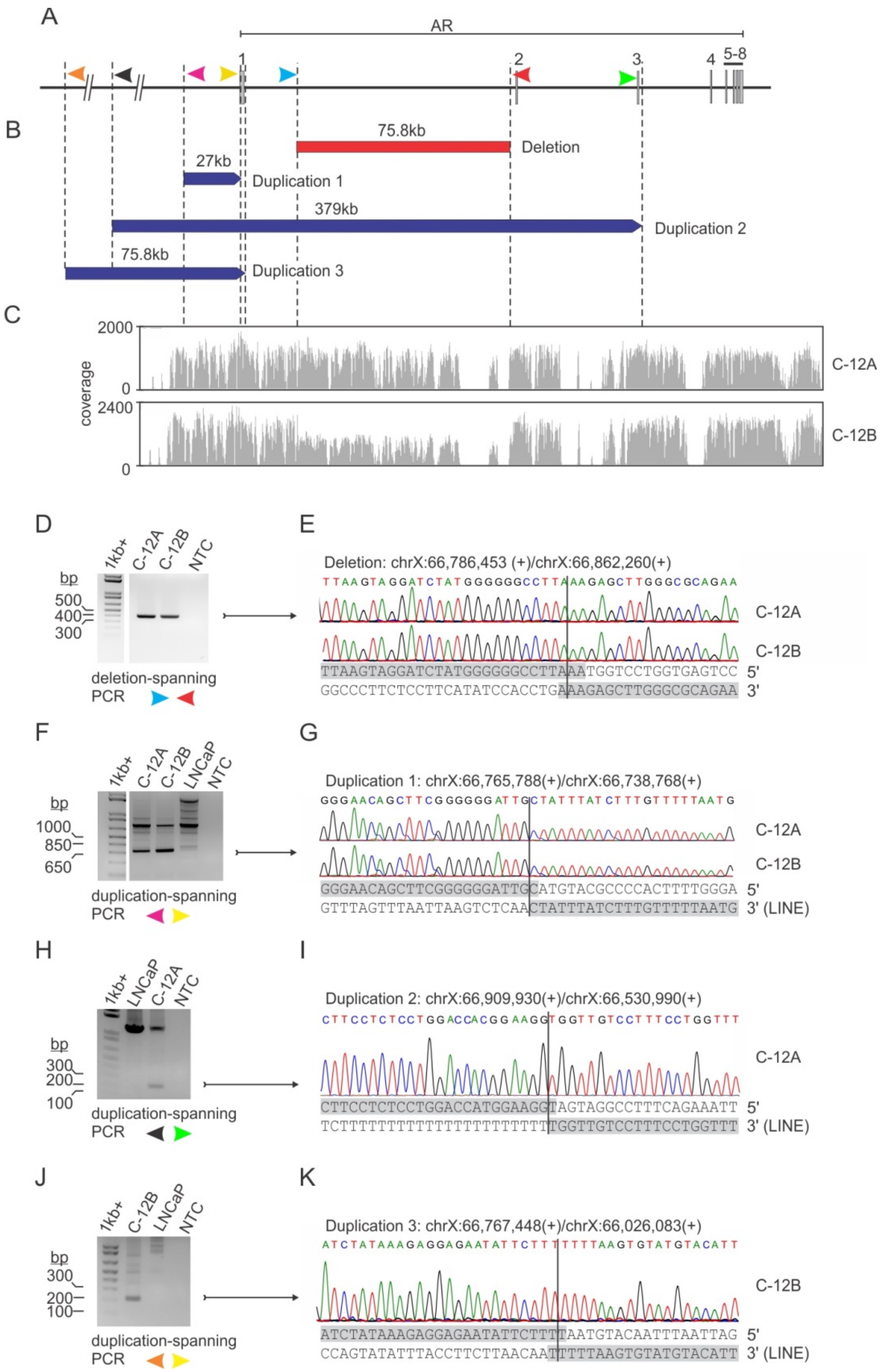

Supplementary Figure 6. ***AR*** **genomic structural rearrangement validation in subject C-12. A and B,** binding locations of PCR primers (colored arrowheads) for validation of the Deletion and Duplications 1, 2, and 3 in tumors C-12A and C-12B. **C,** AR DNA-seq coverage for tumors C-12A and C-12B was assessed by visualizing BAM files of mapped AR DNA-seq reads in Integrative Genomics Viewer. **D,** PCR was performed with a primer set designed for isolation of the Deletion (illustrated in A and B) using DNA from tumors C-12A and C-12B as well as no template control (NTC) as negative control.

Supplementary Figure 6 legend, continued.

E, Sanger sequencing analysis of the tumor C-12A and C-12B Deletion PCR products. The genomic sequences corresponding to the 5' and 3' breakpoints are indicated, with gray shading representing the sequences contained in the break fusion junction signature. **F,** PCR was performed with a primer set designed for isolation of Duplication 1 (illustrated in A and B) using DNA from tumors C-12A and C-12B as well as LNCaP or no template control (NTC) as negative controls. **G,** Sanger sequencing analysis of the tumor C-12A and C-12B Duplication 1 PCR products. The genomic sequences corresponding to the 5' and 3' breakpoints are indicated, with gray shading representing the sequences contained in the break fusion junction signature. The 3' breakpoint sequence is located in a LINE-1 element **H,** PCR was performed with a primer set designed for isolation of Duplication 2 (illustrated in A and B) using DNA from tumor C-12A as well as LNCaP or no template control (NTC) as negative controls. **I,** Sanger sequencing analysis of the tumor C-12A Duplication 2 PCR product. The genomic sequences corresponding to the 5' and 3' breakpoints are indicated, with gray shading representing the sequences contained in the break fusion junction signature. The 3' breakpoint sequence is located in a LINE-1 element. **J,** PCR was performed with a primer set designed for isolation of Duplication 3 (illustrated in A and B) using DNA from tumor C-12A as well as LNCaP or no template control (NTC) as negative controls. **K,** Sanger sequencing analysis of the tumor C-12B Duplication 3 PCR product. The genomic sequences corresponding to the 5' and 3' breakpoints are indicated, with gray shading representing the sequences contained in the break fusion junction signature. The 3' breakpoint sequence is located in a LINE-1 element.

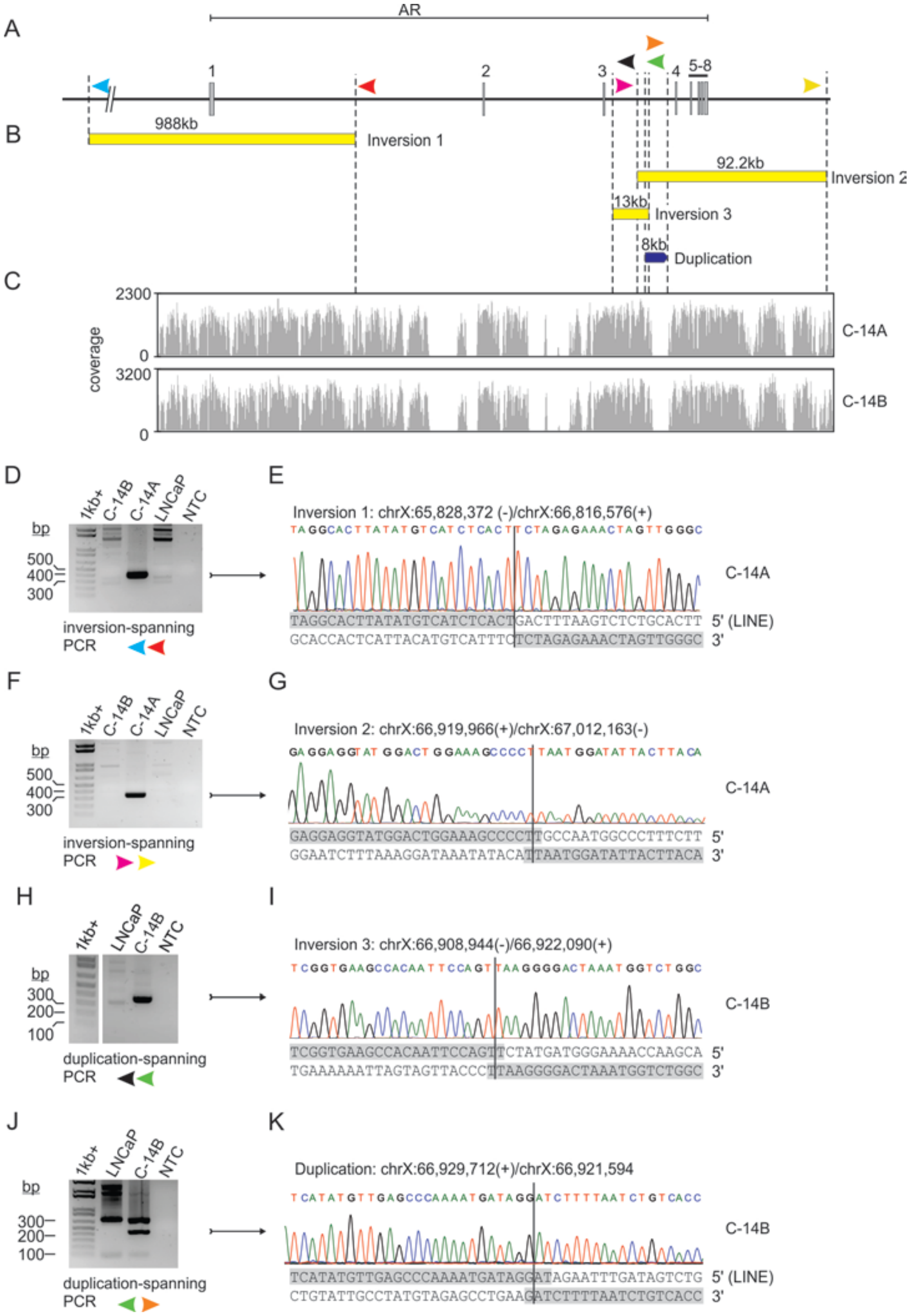

Supplementary Figure 7. *AR* genomic structural rearrangement validation in subject C-14. A and B, binding locations of PCR primers (colored arrowheads) for validation of Inversions 1, 2, and 3, and the Duplication in tumors C-14A and C-14B. **C,** AR DNA-seq coverage for tumors C-14A and C-14B was assessed by visualizing BAM files of mapped AR DNA-seq reads in Integrative Genomics Viewer. **D,** PCR was performed with a primer set designed for isolation of Inversion 1 (illustrated in A and B) using DNA from tumors C-14A and C-14B as well as LNCaP DNA and no template control (NTC) as negative controls. **E,** Sanger sequencing analysis of the tumor C-14A Inversion 1 PCR product. The genomic sequences corresponding to the 5' and 3' breakpoints are indicated, with gray shading representing the sequences contained in the break fusion junction signature. The 5' breakpoint sequence is located in a LINE-1 element **F,** PCR was performed with a primer set designed for isolation of Inversion 2

Supplementary Figure 7 legend, continued.

(illustrated in A and B) using DNA from tumors C-14A and C-14B as well as LNCaP or no template control (NTC) as negative controls. **G,** Sanger sequencing analysis of the tumor C-14A Inversion 1 PCR product. The genomic sequences corresponding to the 5' and 3' breakpoints are indicated, with gray shading representing the sequences contained in the break fusion junction signature. **H,** PCR was performed with a primer set designed for isolation of Inversion 3 (illustrated in A and B) using DNA from tumor C-14B as well as LNCaP or no template control (NTC) as negative controls. **I,** Sanger sequencing analysis of the tumor C-14B Inversion 3 PCR product. The genomic sequences corresponding to the 5' and 3' breakpoints are indicated, with gray shading representing the sequences contained in the break fusion junction signature. **J,** PCR was performed with a primer set designed for isolation of the Duplication (illustrated in A and B) using DNA from tumor C-14B as well as LNCaP or no template control (NTC) as negative controls. **K,** Sanger sequencing analysis of the tumor C-14B Duplication PCR product. The genomic sequences corresponding to the 5' and 3' breakpoints are indicated, with gray shading representing the sequences contained in the break fusion junction signature. The 5' breakpoint sequence is located in a LINE-1 element.

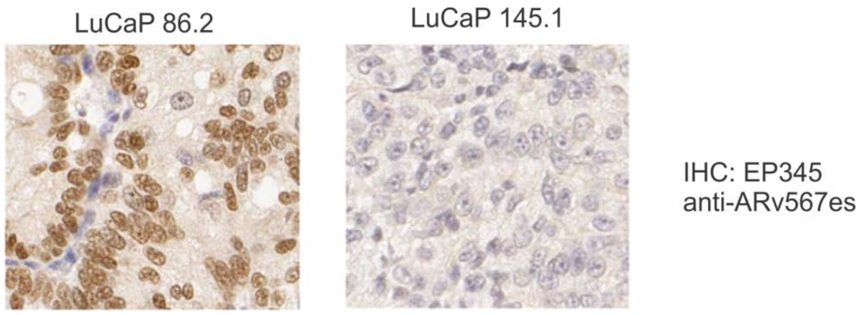

Supplementary Figure 8. Immunohistochemistry with ARv567es rabbit monoclonal antibody EP345. Xenograft tissues positive (LuCaP 86.2) or negative (LuCaP 145.1) for ARv567es were subjected to IHC with EP345.

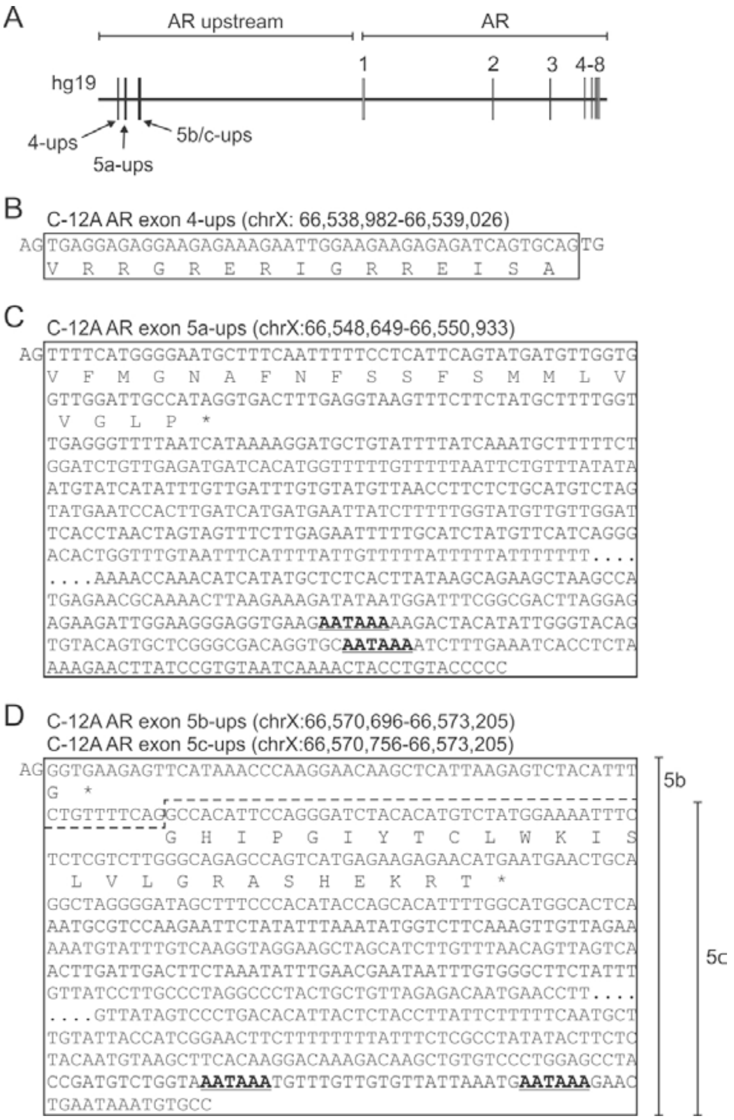

Supplementary Figure 9. Exons expressed in the context of AR-Vs in tumor C-12A. A, location of AR exons 4-ups, 5a-ups, and 5b/c-ups in the context of the non-rearranged hg19 reference genome architecture. Exon nomenclature utilizes numbering/lettering to reflect position in the AR transcript and "ups" to reflect the genomic location upstream of *AR*. **B,** genomic sequence of AR exon 4-ups with canonical flanking intronic AG/TG dinucleotides. Translation in the context of the reading frame of the expressed AR-V is indicated. **C,** genome sequence of 3' terminal exon 5a-ups with a canonical upstream intronic AG dinucleotide. Translation in the context of the reading frame of the expressed AR-V is indicated. Canonical polyadenylation signals (AATAAA) located upstream of RNA-seq coverage drop-off are underlined and bold. **D,** genome sequences of 3' terminal AR exons 5b- or 5c-ups with the two splice acceptor sites containing canonical upstream intronic AG dinucleotides. Two possible translation reading frames for expressed AR-Vs are indicated based upon usage of the exon 5b or 5c splice acceptor site. Canonical polyadenylation signals (AATAAA) located upstream of RNA-seq coverage drop-off are underlined and bold.

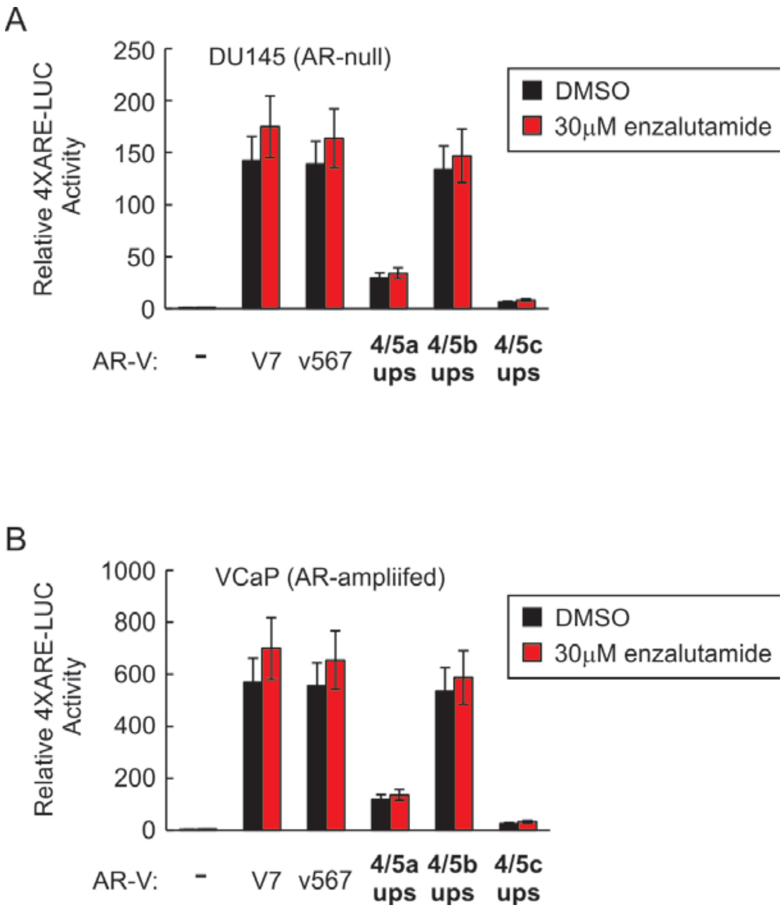

Supplementary Figure 10. Luciferase assays in DU145 and VCaP cells with AR-Vs specific to tumor C-12A. A and B, AR-null DU145 prostate cancer cells (A) and AR amplified VCaP prostate cancer cells (B) were transfected with an androgen response element (ARE)-driven luciferase reporter and expression vectors encoding AR-Vs as indicated. AR-Vs encoded by splicing of novel AR upstream exons from tumor C-12A are indicated in bold. Cells were treated with 30μM enzalutamide (enz) as, or DMSO (vehicle control) as indicated and subjected luciferase assay. Data represent mean +/- S.E. from three biological replicate experiments, each performed in duplicate (n = 6).

A

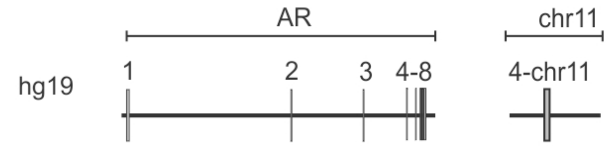

B

C-9A AR exon 4-chr11 (chr11:79,400,501-79,403,211)

AG TTGGAAAAACGAAGGCTCAGAGACCCTGGATCACCCAGTGAGTAAGCAGC
V G K T K A Q R P W I T Q *
ATGCCCAGGTCTAGCATACCATTCTCCTGCTTTCAGCTCTTGTATGCTAT
CCACATGCTTACAGCATGCTTGGACAATGCAGAAACTCAGCTTTGGTACT
TTGGGTAATCTGTTCATCTGTGAAATGGGATAACTCATGCTGATCTACGG
ACAAAAATAACTGTAAGGATGACATGAAATGATGCAGATAGCAGGCACTC
AAAAAACAGAGCTCTATCAACATTCCTGCTTCCTCTCAATGACTGGCACT
GAACCGTCCTTAACACCCCATTTCTAAGCCAAGAGATTCTTCAAGACCAA
GCCAAGTCTGCCGTGCCACTGTCCCCCGCAGGAGCCTCTGGCTTCA....
....TGAGTATAAAATTTCCATCATCGTAGAATGAGAAATGCCTGAAAGC
TGTTAATATACCTACCTTTCTGGACTCTTATTCTGATTCTGTGTCTTTAA
AACACGATGTGGACAGAAAAATCAGAAAATTTGGGGTGAGAAAGGAAATA
ATGAGGAAAAAGAGAAGAAAAGCTCTGCCTCCAACTTTTGCTTTGGTCTT
GTGTAAACCTGTTATCATCTTTGGGTCGGAGATTTCTCTTC**AATAAAATA**
AAATAGATGAACTAA

Supplementary Figure 11. An exon located in chromosome 11 expressed in the context of an AR-V in tumor C-9A. A, location of AR exon 4-chr11 in the context of the non-rearranged hg19 reference genome architecture. Exon nomenclature utilizes numbering to reflect position in the AR transcript and "chr11" to reflect the genomic origin being chromosome 11. **B,** genomic sequence of AR exon 4-chr11 with a canonical upstream intronic AG dinucleotide. Translation in the context of the reading frame of the expressed AR-V is indicated. Canonical polyadenylation signals (AATAAA) located upstream of RNA-seq coverage drop-off are underlined and bold.

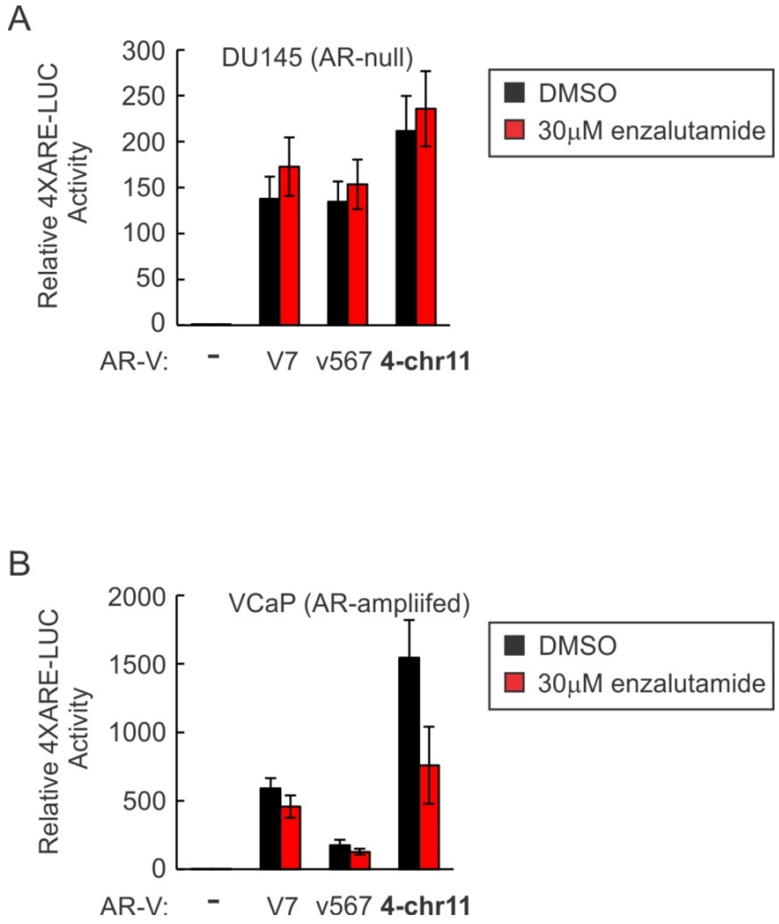

Supplementary Figure 12. Luciferase assays in DU145 and VCaP cells with AR-Vs specific to tumor C-9A. A and B, AR-null DU145 prostate cancer cells (A) and AR amplified VCaP prostate cancer cells (B) were transfected with an androgen response element (ARE)-driven luciferase reporter and expression vectors encoding AR-Vs as indicated. An AR-V encoded by splicing of a novel 3' terminal exon on chromosome 11 in tumor C-9A is indicated in bold. Cells were treated with 30μM enzalutamide (enz) as, or DMSO (vehicle control) as indicated and subjected luciferase assay. Data represent mean +/- S.E. from three biological replicate experiments, each performed in duplicate (n = 6).

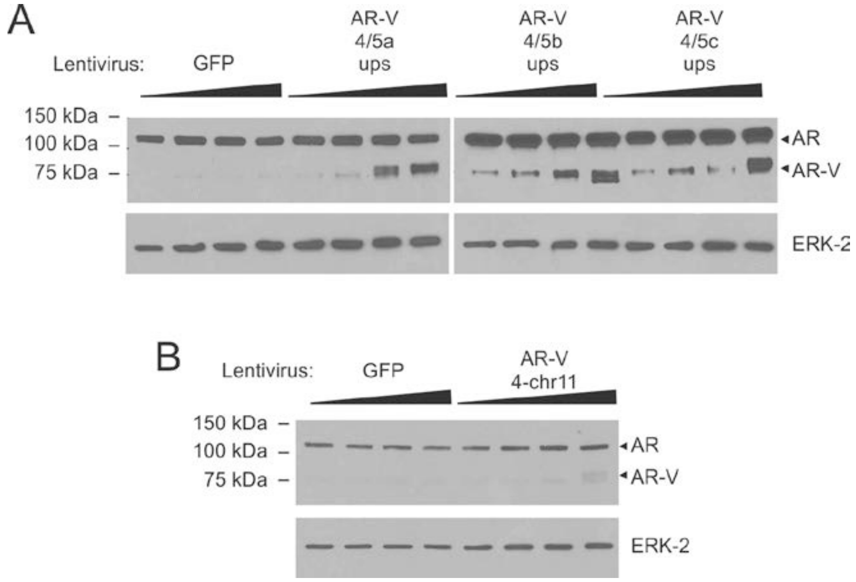

Supplementary Figure 13. AR and ERK-2 western blots of lentivirus-infected LNCaP cells. A, LNCaP cells were infected with a range of titers of lentivirus encoding GFP (control) or AR-Vs from tumor C-12A and subjected to western blot with antibodies specific for the AR NTD and ERK-2. **B,** LNCaP cells were infected with a range of titers of lentivirus encoding GFP (control) or the AR-V from tumor C-9A and subjected to western blot with antibodies specific for the AR NTD and ERK-2.

A

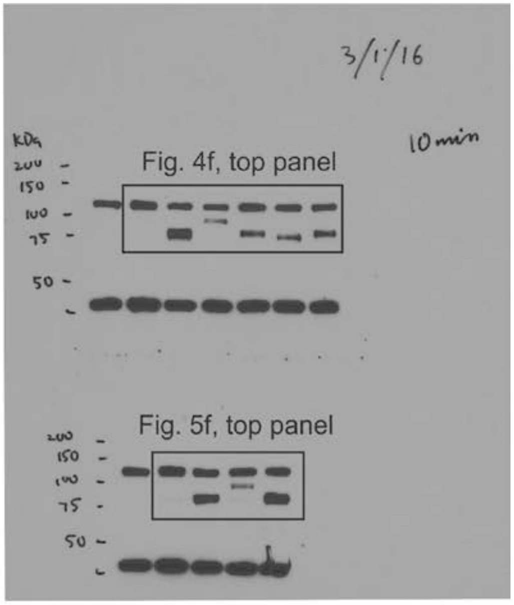

B

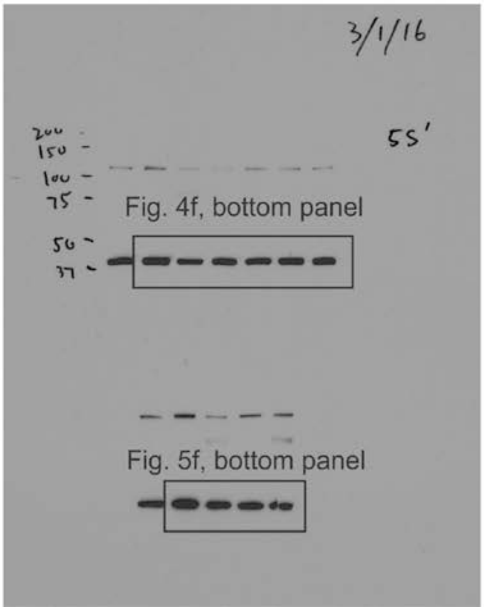

Supplementary Figure 14. Full (uncropped) blots from Figs. 4 and 5. A, Uncropped western blots probed simultaneously with antibodies specific for the AR NTD and ERK-2 and exposed to film for 10 minutes. The areas cropped for display in Figs. 4f and 5f are indicated. **B,** Uncropped western blots processed as in (A) and exposed to film for 5 seconds. The area cropped for display in Figs. 4f and 5f are indicated.

Supplementary Table 1: Features of New Agilent SureSelect AR Capture Panel

**Feature**	**2X Tiled High-Stringency Panel (old)**	**5X Tiled Mod-Stringency Panel (new)**
Total Probes	1381	16468
Probes Overlapping AR	1357	4623
AR Bases Covered by Probes	90389	128137
% AR Bases Covered by Probes	48.40%	68.70%
Uniquely covered AR Bases vs. Other Panel	82	37830

Supplementary Table 2: CPRC Tissue Metastatic Locations

Sample	Tumor Location	% Cancer in Adjacent Tissue Section
C-1A	liver met	60
C-1B	lymph node met	90
C-2A	lymph node met	80
C-2B	mesentery lymph node met #1	80
C-3A	mesenteric lymph node met	95
C-3B	pretrachial lymph node met	99
C-4A	right iliac lymph node met	95
C-4B	peri bile duct lymph node met	70
C-5A	lymph node met	95
C-5B	iliac lymph node met	80
C-6A	supra kidney LN met	90
C-6B	para aortic LN met	90
C-7A	pre aortic lymph node met #2	90
C-7B	pre aortic lymph node met #3	95
C-8A	peri aortic lymph node met	100
C-8B	lung met	95
C-9A	left periaortic lymph node met	80
C-9B	aortic bifurcation lymph node	90
C-10A	aortic lymph node met	80
C-10B	left adrenal met	70
C-11A	right diaphragm met	95
C-11B	right iliac lymph node	85
C-12A	liver met 1	70
C-12B	liver met 2	90
C-13A	peri aortic lymph node met	30
C-13B	bladder met	100
C-14A	right iliac pelvic lymph node met	90
C-14B	liver met	80
C-15A	abdominal lymph node met 2	70
C-15B	left peri aortic lymph node met	80

Supplementary Table 3: Clinical Data for Rapid Autopsy Subjects

Rapid Autopsy Subject #	Gleason	PSA at Diagnosis	Age at Diagnosis	Age at Death (years)	Survival from Diagnosis (years)	Prostatectomy	Androgen Deprivation Therapy	Androgen Deprivation Therapy (Type)	Androgen Independence (years)	Androgen Ablation to Death (years)	First Bone Metastasis Age (years)	Bone Metastasis Delay from Diagnosis (years)	Survival from First Bone Metastasis (years)	Duration Ketoconazole (months)
C-1	4+3	161	80	84	4.33	N	Y	Flutamide plus Lupron	1.91	4.2	80	0.0	4.3	N
C-2	9	NA	77	84	6.93	N	Y	B orchiectomy followed by Flutamide plus Lupron	5.84	2.9	81	4.0	2.9	N
C-3	3+5	11.5	66	70	4.48	N	Y	Casodex plus Zoladex	1.11	4.0	70	4.1	0.4	N
C-4	UNK	403.5	63	65	1.72	N	Y	Lupron, Finasteride, Flutamide	0.68	1.5	63	0.0	1.7	N
C-5	4+5	245	43	47	3.97	Y	Y	Lupron	1.05	4.0	46	3.5	0.5	6.05
C-6	UNK	UNK	53	63	9.44	Y	Y	Lupron, Casodex	6.33	8.2	59	5.9	3.5	10.06
C-7	4+3	10	74	86	11.76	N	Y	Casodex	9.97	11.4	84	10.1	1.7	11.28
C-8	3+4	UNK	73	83	10.49	Y	Y	Casodex plus Lupron	9.24	6.6	83	9.7	0.8	N
C-9	3+3	10	64	72	8.05	Y	Y	Lupron/Casodex	6.80	4.7	N/A	N/A	N/A	1.91
C-10	5+4	268	54	71	16.37	N	Y	Orchiectomy, Flutamide, Casodex	4.86	15.4	63	8.5	7.9	3.88
C-11	3+4	3.3	65	76	11.39	N	Y	Casodex/Lupron	3.07	8.9	74	9.0	2.4	2.76
C-12	4+5	11.3	71	77	6.32	Y	Y	Casodex, Lupron, Dutasteride, Nilutamide, Abiraterone	2.33	5.9	73	2.4	3.9	3.42
C-13	UNK	50	59	61	1.56	N	Y	Zoladex, Casodex, Abiraterone	UNK	1.4	60	0.2	1.4	N
C-14	4+5	105	43	45	1.35	N	Y	Lupron, Casodex, Abiraterone, Enzalutamide	0.65	1.3	43	0.0	1.3	N
C-15	3+3	<10	70	82	11.58	N	Y	Vantas implants, Lupron, Casodex, Abiraterone, Enzalutamide	8.93	7.0	79	9.0	2.2	N

Abbreviations: N = no; UNK = unknown; N = no; N/A = not applicable

Supplementary Table 3, cont'd: Clinical Data for Rapid Autopsy Subjects

Rapid Autopsy Subject #	Duration Ketoconazole (months)	Duration DES/estrogen (months)	DES+Ketoconazole	Duration Corticosteroid (months)	Duration Taxotere (months)	Duration Taxol (months)	Duration Carboplatin (months)	Duration Estramustine (month)	Duration Mitoxantrone (months)	Duration Abiraterone (months)	Duration Enzalutamide (months)	Bisphosphonates	Duration Bisphosphonates (months)	Vaccine	Type of Vaccine	Final Serum PSA (ng/mL)
C-1	N	N	N	4.54	N	N	N	N	4.54	N	N	Pamidronate	12.59	N		128.70
C-2	N	N	N	N	N	N	N	N	N	N	N	Zometa	6.21	N		2038.90
C-3	N	5.65	N	N	3.78	3.42	3.42	3.78	N	N	N	Pamidronate	22.09	N		325.00
C-4	N	N	N	6.90	UNK	N	N	N	6.90	N	N	Pamidronate	8.68	N		396.00
C-5	6.05	2.24	Y	6.28	N	N	N	N	N	N	N	N	N	N		201.70
C-6	10.06	3.52	Y	5.75	9.57	2.79	2.79	N	3.78	N	N	Alendronate/ Zometa	23.44	N		3919.00
C-7	11.28	N	N	N	N	N	N	N	N	N	N	N	N	Y	GVAX	1508.40
C-8	N	3.94	N	N	N	10.91	10.91	N	N	N	N	Zometa	7.50	N		114.83
C-9	1.91	N	N	N	N	N	N	N	N	N	N	N	N	N		298.70
C-10	3.88	13.77	N	36.26	10.36	N	N	N	N	N	N	Zometa	63.12	Y	GVAX	735.97
C-11	2.76	7.00	N	54.80	38.17	13.77	13.77	N	5.52	N	N	Zometa	96.19	N		5690.80
C-12	3.42	N	N	29.91	8.38	N	6.25	N	N	2.76	N	N	N	N		1181.75
C-13	N	N	N	UNK	UNK	N	N	N	N	3.98	N	Pamidronate	UNK	N		30.60
C-14	N	N	N	7.89	2.93	N	N	N	N	2.99	1.35	N	N	N		1949.44
C-15	N	N	N	6.21	6.67	N	N	N	N	2.53	4.01	Zomata	22.52	Y	PROVENGE	1158.53

Abbreviations: N = no; UNK = unknown; N = no; N/A = not applicable

Supplementary Table 4: *AR* Coverage and Copy Number

Tumor ID	Tumor Type	Mean Per-Base Read Coverage of AR	AR Copy Number	AR bases with zero coverage	AR bases with coverage
C-1A	met	702	1	29584	157004
C-1B	met	556	1	26532	160056
C-2A	met	283	1	23892	162696
C-2B	met	419	1	30043	156545
C-3A	met	1135	4	27066	159522
C-3B	met	1087	4	27433	159155
C-4A	met	949	4	27859	158729
C-4B	met	867	4	23899	162689
C-5A	met	426	1	29277	157311
C-5B	met	488	1	30307	156281
C-6A	met	456	1	30179	156409
C-6B	met	427	1	29962	156626
C-7A	met	1134	5	26186	160402
C-7B	met	874	4	30253	156335
C-8A	met	553	1	29035	157553
C-8B	met	448	1	27761	158827
C-9A	met	382	1	26069	160519
C-9B	met	528	1	29054	157534
C-10A	met	587	1	28566	158022
C-10B	met	583	1	30049	156539
C-11A	met	714	2	29043	157545
C-11B	met	466	1	29710	156878
C-12A	met	732	2	29848	156740
C-12B	met	849	3	26242	160346
C-13A	met	313	1	27651	158937
C-13B	met	469	1	27916	158672
C-14A	met	981	4	27411	159177
C-14B	met	1293	9	27110	159478
C-15A	met	543	1	28929	157659
C-15B	met	531	2	30460	156128

Supplementary Table 4, cont'd: *AR* Coverage and Copy Number

P-1	TURP	927	2	27323	159265
P-3	TURP	743	1	27732	158856
P-14	TURP	993	2	26364	160224
P-17	TURP	789	1	26852	159736
P-18	TURP	553	1	25549	161039
P-25	TURP	419	1	29005	157583
P-2	prostatectomy	418	1	28329	158259
P-4	prostatectomy	389	1	26825	159763
P-5	prostatectomy	419	1	28472	158116
P-6	prostatectomy	207	1	25267	161321
P-7	prostatectomy	419	1	28494	158094
P-8	prostatectomy	382	1	29434	157154
P-9	prostatectomy	344	1	28462	158126
P-10	prostatectomy	363	1	29747	156841
P-11	prostatectomy	392	1	29499	157089
P-12	prostatectomy	304	1	31210	155378
P-13	prostatectomy	377	1	31221	155367
P-15	prostatectomy	396	1	30248	156340
P-16	prostatectomy	438	1	28743	157845
P-19	prostatectomy	426	1	27374	159214
P-20	prostatectomy	454	1	30281	156307
P-21	prostatectomy	351	1	30123	156465
P-22	prostatectomy	351	1	27060	159528
P-23	prostatectomy	420	1	31253	155335
P-24	prostatectomy	371	1	30225	156363
P-26	prostatectomy	345	1	27664	158924
P-27	prostatectomy	357	1	29139	157449

Abbreviations: met = metastasis; TURP = transurethral resection of the prostate

Supplementary Table 5: *AR* Coding Single Nucleotide Variants

Tumor ID	chromosome position	wild-type base	variant base	AR amino acid position	wild-type amino acid	variant amino acid	reads with wild-type base	reads with variant base	variant allele %
C-1A	chrX: 66,943,552	A	G	878	T	A	597	720	54.67%
C-1B	chrX: 66,943,552	A	G	878	T	A	123	702	85.09%
C-8A	chrX: 66,931,463	T	A	702	L	H	579	354	37.86%
C-8B	chrX: 66,931,463	T	A	702	L	H	487	311	38.97%
C-10A	chrX: 66,943,552	A	G	878	T	A	211	845	79.94%
C-10B	chrX: 66,943,552	A	G	878	T	A	298	716	70.54%

Supplementary Table 6: Clinical Data for TURP and Prostatectomy Specimens

Patient	Specimen	Year	Age at Diagnosis	Age at Surgery	Race	Gleason Score	PSA	prior ADT	CRPC	metastases	current therapy	prior therapies
P-1	TURP	2013	53	58	Caucasian	4+5=9	9.6	yes	yes	yes	docetaxel	bicalutamide, lupron, RT
P-3	TURP	2013	64	72	Caucasian	4+5=9	5.9	yes	yes	yes	degarelix	lupron, bicalutamide
P-14	TURP	2014	49	55	Caucasian	5+5=10	16.3	yes	yes	yes	docetaxel	lupron, bicalutamide, enzalutamide, RT, provenge
P-17	TURP	2014	64	73	Caucasian	5+5=10	5.9	yes	yes	yes	docetaxel	lupron, bicalutamide, tamoxifen, avodart
P-18	TURP	2015	63	67	hispanic	4+5=9	8.9	yes	yes	no	lupron	bicalutamide, lupron
P-25	TURP	2015	66	71	African American	4+4=8	4.6	yes	yes	no	lupron	bicalutamide, lupron
P-2	prostatectomy	2013	64	64	Indian	7	10.3	no	no	no	none	none
P-4	prostatectomy	2013	56	57	Caucasian	6	5.7	no	no	no	none	none
P-5	prostatectomy	2013	58	58	African American	9	4.6	no	no	no	none	none
P-6	prostatectomy	2013	66	66	Caucasian	8	8.9	no	no	no	none	none
P-7	prostatectomy	2013	61	61	Caucasian	7	8.6	no	no	no	none	none
P-8	prostatectomy	2013	66	67	hispanic	7	11.6	no	no	no	none	none
P-9	prostatectomy	2014	63	63	Caucasian	7	4.4	no	no	no	none	none
P-10	prostatectomy	2014	62	62	African American	8	3	no	no	no	none	none
P-11	prostatectomy	2014	54	54	Caucasian	7	8.9	no	no	no	none	none
P-12	prostatectomy	2014	55	55	Caucasian	7	9.6	no	no	no	none	none
P-13	prostatectomy	2014	59	59	African American	9	14.3	no	no	no	none	none
P-15	prostatectomy	2014	58	61	hispanic	6	14.6	no	no	no	none	none
P-16	prostatectomy	2014	62	62	Caucasian	8	17	no	no	no	none	none
P-19	prostatectomy	2015	64	64	African American	7	12	no	no	no	none	none
P-20	prostatectomy	2015	65	65	hispanic	8	8.3	no	no	no	none	none
P-21	prostatectomy	2015	62	63	Caucasian	8	4.6	no	no	no	none	none
P-22	prostatectomy	2015	58	58	African American	7	5.9	no	no	no	none	none
P-23	prostatectomy	2015	53	53	hispanic	7	6.8	no	no	no	none	none
P-24	prostatectomy	2015	57	58	Caucasian	8	5.6	no	no	no	none	none
P-26	prostatectomy	2015	59	59	Caucasian	8	4.2	no	no	no	none	none
P-27	prostatectomy	2015	61	63	hispanic	7	11.6	no	no	no	none	none

Abbreviations: ADT, androgen deprivation therapy; CRPC, castration-resistant prostate cancer; PSA, prostate specific antigen; RT, radiation therapy; TURP = transurethral resection of the prostate

Supplementary Table 7: Oligonucleotide Primers for *AR*-GSR Validation

Tumor ID	AR Rearrangement	Orientation	Primer sequence	Genomic coordinates of primer binding site	
C-4A	Deletion 1	F	ACGCAAAGCACAGGACTAGT	ChrX:66803819	ChrX:66803838
		R	TCAAGTGAGAGTCCTGCAGC	ChrX:66816619	ChrX:66816600
	Deletion 2	F	CACACAAGCTAAATGTCCTTGC	ChrX:66754887	ChrX:66754908
		R	AGTACTGCTTTAGCTTGGCTGT	ChrX:66843323	ChrX:66843344
C-6A	Deletion 1	F	AGGCTACTTCAGAGATTGGGC	ChrX:66934661	ChrX:66934681
		R	CCCCACAGGGAACCATCTAC	ChrX:66942571	ChrX:66942552
	Deletion 2	F	ACTGAACTTCTATGTGCCGC	ChrX:66939869	ChrX:66939888
		R	TCCCAGAAAGGATCTTGGGC	ChrX:66943651	ChrX:66943632
	Duplication	F	GGTGGGGGTCAAGTCTGTG	ChrX:66942571	ChrX:66942589
		R	GCTTGGTGTTGAAGAGATCAGC	ChxX:66939766	ChxX:66939745
C-6B	SAME AS C-6A		SAME AS C-6A		
C-6C	SAME AS C-6A		SAME AS C-6A		
C-7A	Deletion	F	GCAAAACCCCACACAAGGAT	ChrX:49150490	ChrX:49150509
		R	AGGGACGGAGGTAGCATCTT	ChrX:66779338	ChrX:66779319
C-7B	SAME AS C-7A		SAME AS C-7A		
C-9A	Translocation	F	ATTGTCTGTTCCCTGCTCCC	ChrX:66909044	ChrX:66909063
		R	ACCCTGGGGAAAGAGGAAGA	Chr11:79397819	Chr11:79397800
C-12A	Deletion	F	CAGCAACTTGTGAAACGCCA	ChrX:66786193	ChrX:66786212
		R	TCCGCCAGATTCCATTCCAC	ChrX:66862447	ChrX:66862428
	Duplication 1	F	GCAACTCCTTCAGCAACAGC	ChrX:66765561	ChrX:66765580
		R	TGACCAAGAACCCAAAAGCA	ChrX:66738794	ChrX:66738813
	Duplication 2	F	TGGTGTTGGTCCCTGTTGAT	ChrX:66909885	ChrX:66909904
		R	ACAAGTCCAGGACCAGACAG	ChrX:66531140	ChrX:66531140
C-12B	Deletion		SAME AS C-12A		
	Duplication1		SAME AS C-12A		
	Duplication3	F	AGATCCACAGCCCCCTACTT	ChrX:66767390	ChrX:66767409
		R	TGGAATGGGGATTGCCAGAG	ChrX:66026233	ChrX:66026214
C-14A	Inversion 1 Right breakpoint	F	TCAGCCTGAAGGACTCTCTTC	ChrX:65828536	ChrX:65828516
		R	TCAAGTGAGAGTCCTGCAGC	ChrX:66816619	ChrX:66816600
	Inversion 2 Left breakpoint	F	CCCCAGGGCTGAAAAGTTAGT	ChrX:66919865	ChrX:66919885
		R	GGAGTAGTGCAGCTAGGCAA	ChrX:67011945	ChrX:67011964
C-14B	Inversion 3 Right breakpoint	F	TCGGTGAAGCCACAATTCCA	ChrX:66908965	ChrX:66908946
		R	GCCCTGAGTGCAAGATGACT	ChrX:66922440	ChrX:66922421
	Duplication	F	CCAACAAGCCCCAGTGAGAT	ChrX:66929502	ChrX:66929521
		R	ATCATCCCTGCCTCCCTTCT	ChrX:66921806	ChrX:66921787

Supplementary Table 8: Oligonucleotide Primers for RT-PCR

Target	Detail	Sequence
AR-V7	F Primer	5'- TGT CGT CTT CGG AAA TGT TAT GA -3'
	R Primer	5'- TCA TTT TGA GAT GCT TGC AAT TG -3'
	TaqMan Probe	6FAM-TCT GGG AGA AAA ATT -MGBNFQ
ARv567es	F Primer	5'- CCTTGCTCTCTAGCCTCAATGAA -3'
	R Primer	5'- CTTGATTAGCAGGTCAAAAGTGAACT -3'
	TaqMan Probe	6FAM-CCT TGC CTG ATT GCG AGA -MGBNFQ

Supplementary Table 9: Oligonucleotides for AR-V Construction

AR-V	Detail	Sequence
1/2/3/4a-ups/5a-ups	cassette F strand	5'-ctagGAGTGAGGAGAGGAAGAGAAAGAATTGGAAGAAGAGAGATCAGTGCAGTTTTCATGGGGAATGCTTTCAATTTTTCCTCATTCAGTATGATGTTGGTGGTTGGATTGCCATAGt
	cassette R strand	5'-ctagaCTATGGCAATCCAACCACCAACATCATACTGAATGAGGAAAAATTGAAAGCATTCCCCATGAAAACTGCACTGATCTCTCTTCTTCCAATTCTTTCTCTTCCTCTCCTCACTC
1/2/3/4a-ups/5b-ups	cassette F strand	5'-ctagGAGTGAGGAGAGGAAGAGAAAGAATTGGAAGAAGAGAGATCAGTGCAGGGTGAt
	cassette R strand	5’-ctagaTCACCCTGCACTGATCTCTCTTCTTCCAATTCTTTCTCTTCCTCTCCTCACTC
1/2/3/4a-ups/5c-ups	cassette F strand	5’ctagGAGTGAGGAGAGGAAGAGAAAGAATTGGAAGAAGAGAGATCAGTGCAGGCCACATTCCAGGGATCTACACATGTCTATGGAAAATTTCTCTCGTCTTGGGCAGAGCCAGTCATGAGAAGAGAACATGAt
	cassette R strand	5’-ctagaTCATGTTCTCTTCTCATGACTGGCTCTGCCCAAGACGAGAGAAATTTTCCATAGACATGTGTAGATCCCTGGAATGTGGCCTGCACTGATCTCTCTTCTTCCAATTCTTTCTCTTCCTCTCCTCACTC
1/2/3/4-chr11	cassette F strand	5'-ctagGAGTTGGAAAAACGAAGGCTCAGAGACCCTGGATCACCCAGTGAt
	cassette R strand	5'-ctagaTCACTGGGTGATCCAGGGTCTCTGAGCCTTCGTTTTTCCAACTC
